# A fast and accurate computational method for the linear-combination-based isotropic periodic sum

**DOI:** 10.1038/s41598-018-30364-2

**Published:** 2018-08-08

**Authors:** Kazuaki Z. Takahashi, Takuma Nozawa, Kenji Yasuoka

**Affiliations:** 1 Research Center for Computational Design of Advanced Functional Materials, National Institute of Advanced Industrial Science and Technology (AIST), Central 2, 1-1-1 Umezono, Tsukuba, Ibaraki 305-8568 Japan; 2 Department of Mechanical Engineering, Keio University, 3-14-1 Hiyoshi, Kohoku-ku, Yokohama, Kanagawa 223-8522 Japan

## Abstract

An isotropic periodic sum (IPS) is a powerful technique to reasonably calculate intermolecular interactions for wide range of molecular systems under periodic boundary conditions. A linear-combination-based IPS (LIPS) has been developed to attain computational accuracy close to an exact lattice sum, such as the Ewald sum. The algorithm of the original LIPS method has a high computational cost because it needs long-range interaction calculations in real space. This becomes a performance bottleneck for long-time molecular simulations. In this work, the combination of an LIPS and fast Fourier transform (FFT) was developed, and evaluated on homogeneous and heterogeneous molecular systems. This combinational approach of LIPS/FFT attained computational efficiency close to that of a smooth particle mesh Ewald while maintaining the same high accuracy as the original LIPS. We concluded that LIPS/FFT has great potential to extend the capability of IPS techniques for the fast and accurate computation of many types of molecular systems.

## Introduction

Molecular dynamics (MD) simulations continue to evolve. Recent advances in computer power and algorithmic developments have made it possible to simulate a wide range of molecular systems with reasonable time and length scales for scientific and industrial applications^1–5^.

The computational accuracy of MD has been supported by established molecular models and intermolecular interaction calculation methods^6–14^. Importantly, the accuracy of long-range interaction calculations strongly affects to the accuracy of MD. Under the periodic boundary conditions (PBCs), long-range interactions are ordinally calculated using truncation or lattice sum methods. The simplicity and calculation efficiency are merits of truncation methods, but truncation of long-range interactions can be serious defects on simulating Lennard-Jones (LJ) liquids^15–17^, aqueous^18–28^, and macromolecular systems^29–43^. The reaction field method^44,45^, the Wolf method and its modifications^46,47^, and the smoothing and/or shifting cutoff methods are kinds of truncation methods that have serious defects for various systems (see above citations). The Ewald sum^48^ is used as the most accurate lattice sum method. It incorporates PBCs using the discrete Fourier transform (DFT), but the reciprocal term is computationally expensive. The particle mesh Ewald (PME) and its modifications^49,50^ are particle-particle/particle-mesh approaches that use fast Fourier transforms (FFT) for the Ewald sum, and the smooth PME (SPME)^50^ is now the *de facto* standard lattice sum method. The SPME perform well for intermediate-sized systems ($N\sim {10}^{3}-{10}^{5}$, where *N* is the number of particles)^51^. However, the SPME does not have a high performance for large systems ($N\sim {10}^{6}$ or larger), because it is difficult for FFT to attain strong scaling on massively parallel machines^52,53^. The Barnes-Hut tree-code^54^ and the fast multipole method^55^ are lattice sum methods that use hierarchical tree structures. These tree-based method can attain stronger scaling than FFT because it does not contain reciprocal space calculations that require all-to-all communications on massively parallel machines^53^. The tree-based method has been applied for long-range interaction calculations of molecular simulations^52,56–70^. The Gaussian split Ewald sum^71^ is a combination between the Ewald sum and Poisson equation solver, and can avoid all-to-all communications on massively parallel machines. Importantly, all the lattice sum methods are potentially subject to the symmetry effect that comes from the lattice-like repetition of the unit cell for PBC. This effect is especially troublesome when simulating macromolecular systems, where conformational distributions are sensitive to image interactions^38,40,72–80^. More research into the lattice sum method is required, and the symmetry effect should be carefully considered.

To avoid the symmetry effect, the isotropic periodic sum (IPS) technique is a promising approach. It was first developed by Wu and Brooks^81^. The IPS has become a powerful technique to reasonably calculate intermolecular interactions for a wide range of molecular systems, including net-charge systems under PBCs. It has been applied to solids^82^, liquids^81,83–86^, solid–liquid^87,88^ and liquid-vapor interfaces^89,90^, liquid crystals^91^, proteins^81,92^, lipids^89,93^, combined quantum mechanics/molecular mechanics methods^94^, and constant pH MD simulations^95–97^. Improved methods have been developed for large-scale systems that exploit the possibility of parallel computing^98,99^.

An extended IPS technique, that is, the linear-combination-based IPS (LIPS) was developed to improve the accuracy of the IPS for both homogeneous and heterogeneous systems^7,100–103^. The LIPS provides periodic reaction fields that can design pseudo pair potentials in the range of extended IPS theory. This pseudo pair potential has the high accuracy that achieves computational results close to an exact lattice sum. For example, in the phase transition of liquid crystal systems, the LIPS and SPME with fine grid spacing are the only techniques that can reasonably estimate the solid–liquid-crystalline phase transition temperature^104^. The LIPS has great potential as one of the best possible approaches to contribute to a further substantial advance in IPS techniques. However, the algorithm of the original LIPS has a high computational cost because it requires long-range interaction calculations in real space. This becomes a performance bottleneck for long-time molecular simulations using the LIPS. Several possible approaches to raise the computational efficiency of the LIPS were suggested in the original paper^100^, but have not been applied.

In the present work, the combination of the LIPS and fast Fourier transform (FFT), that is, LIPS/FFT, is developed as a substantial advance in the computational efficiency of the LIPS for intermediate-sized systems $(N\sim {10}^{3}\,\mbox{--}\,{10}^{5})$. The performance of LIPS/FFT is evaluated on homogeneous and heterogeneous polar molecular systems. LIPS/FFT attains computational efficiency close to the SPME while maintaining the high accuracy of the LIPS. We conclude that LIPS/FFT has great potential to extend the capability of IPS techniques for the fast and accurate computation of many types of molecular systems.

## Methodology

### LIPS/FFT method

In the LIPS method, pseudo pair potentials are designed using extended IPS theory, which provides periodic reaction fields^100,101^. Several types of pseudo pair potentials have been developed^100,101^, and these can be expressed as the following equation generally:

1 $${u}_{{\rm{LIPS}}}(r,{R}_{{\rm{c}}})=u(r)+{\varphi }(r,{R}_{{\rm{c}}}),$$

where *u*_LIPS_ is the LIPS pseudo pair potential; *r* is the interaction distance; *R*_c_ is the cutoff radius of the LIPS, which is closely related to the production of periodic reaction fields; *u* is an original pair potential, such as Coulomb interaction; and *ϕ* is the effective potential from the periodic reaction field. The LIPS pseudo pair potential has high accuracy. The previous paper demonstrated that one of the pseudo pair potentials, LIPS-SW, had the same accuracy as the SPME with fine grid spacing (less than 0.1 nm), for the estimation of the solid–liquid-crystalline phase transition temperature^104^. To attain high accuracy, the LIPS potentials require a large ${R}_{{\rm{c}}}$ condition *R*_c_ = *L*/2, where *L* is the length of the longest side of the simulation box. This means long-range interaction calculations in real space, and becomes a performance bottleneck for MD simulations using the LIPS. To avoid the aforementioned difficulty and attain advanced computational efficiency, the combination of the LIPS and FFT is developed as follows: (i) The original LIPS pair potential is divided into short-range, long-range and boundary pair potentials *u*_S_, *u*_L_ and *u*_B_, respectively. For the exact implementation within CHARMM^105–107^ (version c40b2), the following expressions are used:

2 $${u}_{{\rm{S}}}(r,{r}_{{\rm{c}}})=\{\begin{array}{ll}{u}_{{\rm{LIPS}}}(r,{r}_{{\rm{c}}})-{u}_{{\rm{LIPS}}}({r}_{{\rm{c}}},{r}_{{\rm{c}}}) & (r < {r}_{{\rm{c}}})\\ 0 & (r > {r}_{{\rm{c}}})\end{array},$$

3 $${u}_{{\rm{L}}}(r,{r}_{{\rm{c}}},{R}_{{\rm{c}}})=\{\begin{array}{ll}{u}_{{\rm{LIPS}}}(r,{R}_{{\rm{c}}})-{u}_{{\rm{S}}}(r,{r}_{{\rm{c}}})-{u}_{{\rm{B}}}(r,{R}_{{\rm{c}}}) & (r < {R}_{{\rm{c}}})\\ 0 & (r > {R}_{{\rm{c}}})\end{array},$$

4 $${u}_{{\rm{B}}}(r,{R}_{{\rm{c}}})=\{\begin{array}{ll}{u}_{{\rm{LIPS}}}({R}_{{\rm{c}}},{R}_{{\rm{c}}}) & (r < {R}_{{\rm{c}}})\\ 0 & (r > {R}_{{\rm{c}}})\end{array}.$$

where *r*_c_ (<*R*_c_) is the short-range cutoff radius introduced for convenience. (ii) LIPS potential energy *U*_LIPS_ is calculated as the summation of the short-range, long-range, and boundary potential energy, *U*_S_, *U*_L_ and *U*_B_, respectively.

5 $${U}_{{\rm{LIPS}}}={U}_{{\rm{S}}}+{U}_{{\rm{L}}}+{U}_{{\rm{B}}}\mathrm{.}$$

(iii) The short-range potential energy *U*_S_ is simply calculated in real space:

6 $${U}_{{\rm{S}}}=\frac{1}{2}\sum _{i}^{N}{q}_{i}\sum _{{r}_{ij} < {r}_{{\rm{c}}}}{q}_{j}{u}_{{\rm{S}}}({r}_{ij},{r}_{{\rm{c}}}),$$

where *r*_*ij*_ is the interaction distance between particles *i* and *j*. (iv) The long-range potential energy is calculated in reciprocal space using FFT. The details are very similar to those of the SPME^50^. For any simulation box, the charge distribution *q* and potential energy distribution Φ can be exactly defined using matrices that contain positional information for point charges. For the advanced calculation efficiency of FFT, the point charges have to be redistributed to predefined grid points. To do this, the following charge array *Q* is introduced:

7 $$Q({k}_{1},{k}_{2},{k}_{3})=\sum _{i}^{N}\sum _{{n}_{1},{n}_{2},{n}_{3}}{q}_{i}{M}_{n}({w}_{1i}-{k}_{1}-{n}_{1}{K}_{1})\times {M}_{n}({w}_{2i}-{k}_{2}-{n}_{2}{K}_{2})\cdot {M}_{n}({w}_{3i}-{k}_{3}-{n}_{3}{K}_{3}),$$

where *k*_1_, *k*_2_, and *k*_3_ are integers that correspond to predefined grid points; *n*_1_, *n*_2_, and *n*_3_ are integers that correspond to the real space summation; ${M}_{n}$ is the cardinal *b*-spline function of the *n*-th order; *w*_1*i*_, *w*_2*i*_, and *w*_3*i*_ are real numbers; and *K*_1_, *K*_2_, and *K*_3_ are positive integers that correspond to the total number of grid points. Thus, the Fourier transform of *q* is approximated by *Q* as follows:

8 $$F(q)({m}_{1},{m}_{2},{m}_{3})\simeq {b}_{1}({m}_{2}){b}_{1}({m}_{2}){b}_{3}({m}_{3})F(Q)({m}_{1},{m}_{2},{m}_{3}),$$

where *F* is the operator of the discrete Fourier transform (DFT); *m*_1_, *m*_2_, and *m*_3_ are integers that correspond to the reciprocal space summation; and

9 $${b}_{i}({m}_{i})=\frac{\exp \mathrm{(2}\pi i(n-\mathrm{1)}{m}_{i}/{K}_{i})}{\sum _{k=0}^{n-2}{M}_{n}(k+\mathrm{1)}\,\exp \mathrm{(2}\pi i{m}_{i}k/{K}_{i})}\mathrm{.}$$

The term Φ for long-range pair potential at the grid points also has to be defined. Thus, the following energy array Φ^*^ is introduced:

10 $${{\rm{\Phi }}}^{\ast }({k}_{1},{k}_{2},{k}_{3})=\sum _{{n}_{1},{n}_{2},{n}_{3}}{u}_{{\rm{L}}}[r({k}_{1}+{n}_{1}{K}_{1},{k}_{2}+{n}_{2}{K}_{2},{k}_{3}+{n}_{3}{K}_{3})-r(1,1,1),{r}_{{\rm{c}}},{R}_{{\rm{c}}}]\mathrm{.}$$

The convolution between $\,\,Q\,$ and Φ^*^, $\,Q\ast {{\rm{\Phi }}}^{\ast }$, is expressed as follows:

11 $$Q\ast {{\rm{\Phi }}}^{\ast }({j}_{1},{j}_{2},{j}_{3})=\sum _{{k}_{1}=0}^{{K}_{1}-1}\sum _{{k}_{2}=0}^{{K}_{2}-1}\sum _{{k}_{3}=0}^{{K}_{3}-1}Q({k}_{1},{k}_{2},{k}_{3})\cdot {{\rm{\Phi }}}^{\ast }({j}_{1}-{k}_{1},{j}_{2}-{k}_{2},{j}_{3}-{k}_{3}),$$

where *j*_1_, *j*_2_, and *j*_3_ are integers. With the fine grid spacing conditions, $\,Q\ast {{\rm{\Phi }}}^{\ast }$ can be used as the approximation of *q*Φ. For advanced computational efficiency, the convolution calculation using FFT is essential. $\,Q\ast {{\rm{\Phi }}}^{\ast }$ can be simply calculated using DFT and inverse DFT (IDFT):

12 $$Q\ast {{\rm{\Phi }}}^{\ast }={F}^{-1}[F(Q\,\ast \,{{\rm{\Phi }}}^{\ast })]={K}_{1}{K}_{2}{K}_{3}\cdot {F}^{-1}[F(Q)\cdot F({{\rm{\Phi }}}^{\ast })],$$

where *F*^−1^ is the operator of IDFT. Therefore, long-range potential energy *U*_L_ can be calculated as follows:

13 $$\begin{array}{rcl}{U}_{{\rm{L}}} & = & \frac{1}{2}\sum _{i}{q}_{i}\sum _{j}{q}_{j}{u}_{{\rm{L}}}=\frac{1}{2}\sum _{i}{q}_{i}\,q{\rm{\Phi }}\\  & \simeq  & \frac{1}{2}\sum _{i}{q}_{i}\sum _{{m}_{1}=0}^{{K}_{1}-1}\sum _{{m}_{2}=0}^{{K}_{2}-1}\sum _{{m}_{3}=0}^{{K}_{3}-1}{F}^{-1}[{b}_{1}{b}_{2}{b}_{3}F(Q\ast {{\rm{\Phi }}}^{\ast })]\\  & = & \frac{1}{2}\sum _{i}{q}_{i}{K}_{1}{K}_{2}{K}_{3}\sum _{{m}_{1}=0}^{{K}_{1}-1}\sum _{{m}_{2}=0}^{{K}_{2}-1}\sum _{{m}_{3}=0}^{{K}_{3}-1}{b}_{1}{b}_{2}{b}_{3}{F}^{-1}[F(Q)\cdot F({{\rm{\Phi }}}^{\ast })]\\  & \simeq  & \frac{1}{2}\sum _{{m}_{1}=0}^{{K}_{1}-1}\sum _{{m}_{2}=0}^{{K}_{2}-1}\sum _{{m}_{3}=0}^{{K}_{3}-1}B{F}^{-1}(Q)[F(Q)\cdot F({{\rm{\Phi }}}^{\ast })],\end{array}$$

where *B* = |*b*_1_|^2^ ⋅ |*b*_2_|^2^ ⋅ |*b*_3_|^2^. The reciprocal long-range force is calculated using the differential of Eq. 13 with respect to position vector of charges *r*_*i*_:

14 $$-{\nabla }_{i}{U}_{{\rm{L}}}=-\,\frac{\partial {U}_{{\rm{L}}}}{\partial {r}_{\alpha i}}\simeq -\,\sum _{{m}_{1}=0}^{{K}_{1}-1}\sum _{{m}_{2}=0}^{{K}_{2}-1}\sum _{{m}_{3}=0}^{{K}_{3}-1}\frac{\partial Q}{\partial {r}_{\alpha i}}{F}^{-1}[BF(Q)\cdot F({{\rm{\Phi }}}^{\ast })],$$

where *r*_*αi*_ is the *α* (=*x*, *y*, *z*) component of the coordinate of molecule *i*, *r*_*i*_. The term ∂*Q*/∂*r*_*αi*_ can be calculated based on Eq. 7 from the property of the *b*-spline functions:

15 $$\frac{d}{dw}{M}_{n}(w)={M}_{n-1}(w)-{M}_{n-1}(w-\mathrm{1),}$$

where *w* is a real number. (v) The boundary potential energy *U*_B_ is simply calculated in real space:

16 $${U}_{{\rm{B}}}=\frac{1}{2}\sum _{i}^{N}{q}_{i}\sum _{{r}_{ij} < {R}_{{\rm{c}}}}{q}_{j}{u}_{{\rm{B}}}({r}_{ij},{R}_{{\rm{c}}}\mathrm{).}$$

The potential energy and force of LIPS/FFT is calculated from the aforementioned five steps. Note that a similar approach was performed by Wu and Brooks for the combination of the IPS and FFT (IPS/DFFT)^92^.

### Simulation conditions

To evaluate the capability of LIPS/FFT on homogeneous and heterogeneous polar molecular systems, MD simulations for bulk water and water-vapor interfacial systems were performed. For a careful evaluation of the effect of IPS techniques on long-range interaction calculations, all the following three simulation conditions were met. (i) LIPS/FFT was applied only for electrostatic interactions. LIPS-SW^101^ and LIPS-5th^100^ were used for the pseudo pair potential of LIPS/FFT. For comparison, IPS/DFFT and the SPME were also applied only for electrostatic interactions. The IPS method for non-polar systems (IPSn)^81^ and polar systems (IPSp)^84^ were used for IPS/DFFT. For all the aforementioned methods, the short-range cutoff radius for real space interaction calculations and the order for *b*-spline interpolations were set to 1.0 nm and 8, respectively. The factor *α* for the SPME^48–50^ was set to 3.71692 nm^−1^, which corresponds to the short-range cutoff radius. For the treatment of Lennard-Jones (LJ) interactions, the shifting/switching functions to the LJ forces were used^105–107^, with the non-bonded cutoff and inner-cutoff equal to 1.0 nm and 0.99 nm, respectively. (ii) The large cutoff radii *R*_c_ less than or equal to *L*/2 were mainly used under cubic simulation boxes as a result of considering previous reports for the symmetry effect^79,80^. Even though IPS techniques are conceptually different from the lattice sum, LIPS/FFT and IPS/DFFT at infinite *R*_c_ is almost equal to the lattice sum. Moreover, these two methods at *R*_c_ > *L*/2 contain at least the nearest copy boxes of PBCs without any processing by IPS techniques. These facts imply the concern that the combination of IPS techniques and FFT cannot avoid the effect from PBCs when requiring large cutoff radii *R*_c_ > *L*/2 for accuracy. For a secure application, therefore, LIPS/FFT and IPS/DFFT should be used at *R*_c_ ≤ *L*/2. (iii) Fine grid spacing less than or equal to 0.1 nm was used. as a result of considering the previous study for liquid-crystalline phase transition phenomena^104^, which demonstrated that even using the SPME, fine grid spacing conditions (less than 0.1 nm) were required for the accurate estimation of the phase transition temperature of macromolecular systems. LIPS/FFT and IPS/DFFT under fine grid spacing conditions should be evaluated for future applications to complex macromolecular systems.

Bulk water systems were simulated with a constant molecular number, volume, and temperature condition^108–110^. The number of water molecules was 6,192, the size of the simulation box was 5.71 nm × 5.71 nm × 5.71 nm, and the temperature was 298.15 K. The extended simple point charge (SPC/E) model^111^ was used for water molecules. The atoms in each water molecule were constrained by the SHAKE algorithm^112^. The Verlet leapfrog integrator^113^ was used with a time step of 2 fs. All simulation systems were equilibrated prior to data acquisition, and the elapsed time after the equilibration was 1 ns.

The water-vapor interfacial systems were simulated with the constant molecular number, volume, and temperature condition. The number of water molecules was 13,500, the size of simulation box was 10.8 nm × 10.8 nm × 10.8 nm, and the temperature was 298.15 K. The SPC/E model was used for water molecules. The atoms in each water molecule were constrained by the SHAKE algorithm. The Verlet leapfrog integrator was used with a time step of 2 fs. All simulation systems were equilibrated prior to data acquisition, and the elapsed time after the equilibration was 15 ns.

All the aforementioned simulations were performed using CHARMM^105–107^ (version c40b2) modified for LIPS/FFT implementation.

## Results and Discussion

**Figure 1:**
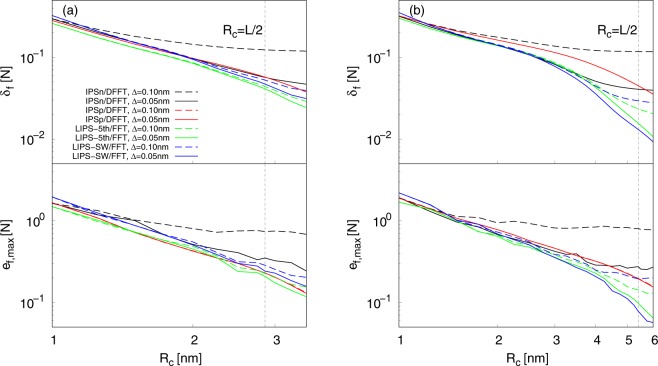
Cutoff radius *R*_c_ dependences of the root-mean-squared deviation of the forces *δ*_*f*_ (top) and the largest error of the forces *e*_*f*,max_ (bottom) for (**a**) bulk water systems and (**b**) water-vapor interfacial systems. *R*_c_ = *L*/2 is also plotted.

To directly evaluate the accuracy of the electrostatic forces calculated by LIPS/FFT, the following two steps were performed: (i) The instantaneous value of the electrostatic forces was calculated for each method using exactly the same coordinates of molecular systems that had been equilibrated using the SPME. Note that the coordinates were not equilibrated using any IPS techniques but using the SPME. (ii) The instantaneous value was compared with that of the SPME using the root-mean-squared deviation of the forces, *δ*_*f*_, and the largest error of the forces, *e*_*f*,max_, defined as follows:

17 $${\delta }_{f}^{2}=\frac{\sum _{i}\sum _{\alpha }|\,{f}_{\alpha ,i}-{f}_{\alpha ,i,{\rm{S}}PME}{|}^{2}}{3N-1},$$

18 $${e}_{f,{\rm{\max }}}=\,\max \,|\,{f}_{\alpha ,i}-{f}_{\alpha ,i,{\rm{S}}PME}|,$$

where *f*_*α*,*i*_ and *f*_*α*,*i*,SPME_ are instantaneous forces calculated by LIPS/FFT and the SPME, respectively. For the calculation of *δ*_*f*_, grid spacing Δ for the SPME was set to 0.05 nm. *δ*_*f*_ and *e*_*f*,max_ for IPS/DFFT were also calculated for comparison. Figure 1(a) shows the *R*_c_ dependences of *δ*_*f*_ (top) and *e*_*f*,max_ (bottom) for bulk water systems for two grid spacing conditions: Δ = 0.1 nm and 0.05 nm. Overall, the *R*_c_ dependences of *δ*_*f*_ and *e*_*f*,max_ were similar to each other method, with few exceptions. However, the results show that LIPS/FFT was in better agreement with the SPME than IPS/DFFT. With increasing *R*_c_, *δ*_*f*_ and *e*_*f*,max_ decreased, except for the case using IPSn/DFFT with Δ = 0.1 nm. This indicates that only IPSn/DFFT with Δ = 0.1 nm barely improved its accuracy with increasing *R*_c_. This very slow decay of *δ*_*f*_ and *e*_*f*,max_ was caused by the cutoff boundary condition of the IPSn pseudo pair potential. This boundary condition was too simple to accurately estimate polar molecular systems^100^. Using finer grid spacings can improved the accuracy of IPSn/DFFT to a level close to the other methods. With decreasing Δ, *δ*_*f*_ and *e*_*f*,max_ decreased, except for the case that used IPSp/DFFT. This indicates that only IPSp/DFFT could not improve its accuracy by choosing fine grid spacing less than 0.1 nm. The IPSp pseudo pair potential included a screening effect from the countercharge modeled on the typical structure of polar molecular systems. This screening effect smeared information for the charge distribution embedded in the fine grids, and then inhibited the accuracy improvement of IPSp/DFFT. Figure 1(b) shows the *R*_c_ dependences of *δ*_*f*_ (top) and *e*_*f*,max_ (bottom) for water-vapor interfacial systems for two grid spacing conditions: Δ = 0.1 nm and 0.05 nm. It is obvious that LIPS/FFT was in much better agreement with the SPME than IPS/DFFT. *δ*_*f*_ and *e*_*f*,max_ started to drastically decrease when *R*_c_ exceeded the thickness of the water slab (∼4 nm), with a few exceptions. This fast decay was because the sphere-like cutoff territory with large *R*_c_ became able to contain the major part of the characteristic structure of molecular systems. For LIPS/FFT, *δ*_*f*_ at *R*_c_ > 4 nm decayed in proportion to ${R}_{{\rm{c}}}^{-3.4}$. For IPSp/DFFT, *δ*_*f*_ decay was clearly slower than it was for LIPS/FFT; *δ*_*f*_ at *R*_c_ > 4 nm decayed in proportion to ${R}_{{\rm{c}}}^{-1.9}$. For IPSn/DFFT, *δ*_*f*_ decay was very slow. Using finer grid spacings could improve the accuracy of IPSn/DFFT; however, the slow decay tendency would still remain. Even using Δ = 0.05 nm, *δ*_*f*_ at *R*_c_ > 4 nm barely decay. With decreasing Δ, *δ*_*f*_ and *e*_*f*,max_ decreased, except for the case using IPSp/DFFT. Similar to the results of bulk water systems, only IPSp/DFFT could not improve its accuracy by choosing fine grid spacing less than 0.1 nm. The screening effect of IPSp inhibited accurate calculation using IPS/DFFT regardless of the homogeneity/heterogeneity of the molecular structure of systems.

**Figure 2:**
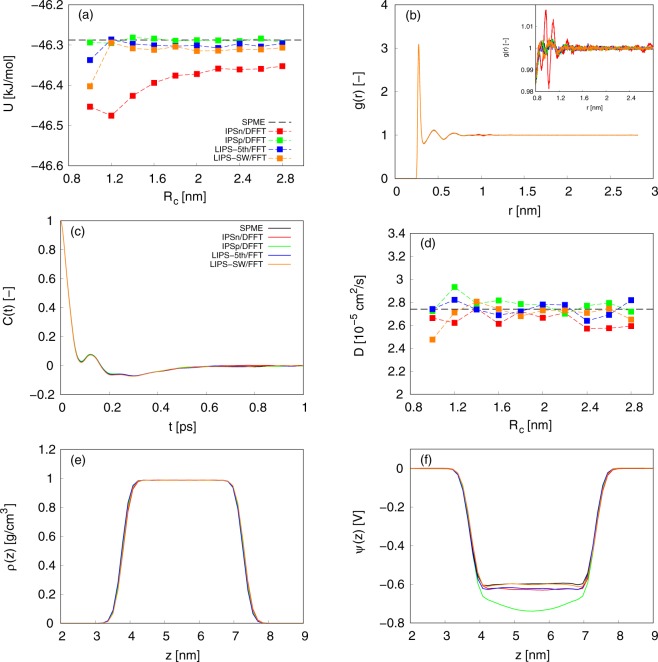
(**a**) Potential energy *U* for bulk water systems at Δ = 0.1 nm. (**b**) Radial distribution function *g*(*r*) for bulk water systems at *R*_c_ = 2.8 nm and ${\rm{\Delta }}=0.1$ nm. (**c**) Velocity auto-correlation function *C*(*t*) for bulk water systems at *R*_c_ = 2.8 nm and Δ = 0.1 nm. (**d**) Diffusion coefficient *D* for bulk water systems at Δ = 0.1 nm. (**e**) Mass density profiles *ϕ*(*z*) for water-vapor interfacial systems at *R*_c_ = *L*/2 and Δ = 0.1 nm. (**f**) Electrostatic potential profiles *ψ*(*z*) for water-vapor interfacial systems at *R*_c_ = *L*/2 and Δ = 0.1 nm.

To evaluate the accuracy of LIPS/FFT for estimating homogeneous/heterogeneous polar molecular systems, some physical properties for bulk water and water-vapor systems were calculated. The potential energy *U* is one of the fundamental thermodynamic properties. Figure 2(a) shows *U* for bulk water systems at Δ = 0.1 nm. In comparison with the results of the SPME, the accuracy for estimating *U* is in the following order: LIPS-5th/FFT (=SPME) = LIPS-SW/FFT = IPSp/DFFT > IPSn/DFFT. This order follows the results of *δ*_*f*_ and *e*_*f*,max_. The radial distribution function *g*(*r*) is a critical property showing local configuration of molecular systems. The conventional expression give,

19 $$g(r)=\frac{V}{4\pi {r}^{2}{\rm{\Delta }}rN(N-\mathrm{1)}}\sum _{i}{n}_{i}(r),$$

where *n*_*i*_(*r*) is the number of molecules in the region between *r* and *r* + Δ*r* from molecule *i*. Figure 2(b) shows the oxygen-oxygen *g*(*r*) for bulk water systems at *R*_c_ = 2.8 nm and Δ = 0.1 nm. In comparison with the results of the SPME, the accuracy for estimating *g*(*r*) is in the following order: LIPS-5th/FFT (=SPME) = LIPS-SW/FFT = IPSp/DFFT > IPSn/DFFT. This order follows the results of *δ*_*f*_ and *e*_*f*,max_. The velocity auto-correlation function *C*(*t*) explains microscopic motion of molecular systems^114^. The conventional expression give,

20 $$C(t)=\frac{\sum _{i}{v}_{i}(t)\cdot {v}_{i}\mathrm{(0)}}{\sum _{i}\,{v}_{i}\mathrm{(0)}\cdot {v}_{i}\mathrm{(0)}},$$

where *v*_*i*_ is the velocity of molecule *i*. Figure 2(c) shows *C*(*t*) for bulk water systems at *R*_c_ = 2.8 nm and Δ = 0.1 nm. All the results were almost same as that of the SPME. The self-diffusion coefficient *D* is one of the representative dynamic properties. *D* can be determined either by the Einstein relation or Green-Kubo formula, which are basically equivalent. Here we used the Einstein relation,

21 $$D=\mathop{\mathrm{lim}}\limits_{t\to \infty }\frac{1}{6Nt}\sum _{i}|{r}_{i}(t)-{r}_{i}{\mathrm{(0)|}}^{2},$$

where *t* is time. The slope of the mean-squared displacement of a diffusing particle in the long-time limit was calculated for the diffusion coefficient. Figure 2(d) shows *D* for bulk water systems at Δ = 0.1 nm. All the results were almost same as that of the SPME. The mass density profile *ρ*(*z*) is one of the fundamental thermodynamic properties showing the mass distribution of heterogeneous systems. Figure 2(e) shows *ρ*(*z*) for water-vapor interfacial systems at *R*_c_ = *L*/2 and Δ = 0.1 nm. All the results were almost same as that of the SPME. The electrostatic potential profile *ψ*(*z*) is well known as one of the properties sensitive to the cutoff radius^84,90,92,100–103^, and thus should be calculated to evaluate the accuracy of the truncation methods. *ψ*(*z*) was calculated using the double integration of the Poisson equation:

22 $$\psi (z)-\psi \mathrm{(0)}=-\,\frac{1}{{\varepsilon }_{0}}{\int }_{0}^{z}{\int }_{0}^{z^{\prime} }{\rho }_{{\rm{c}}}(z^{\prime\prime} )dz^{\prime\prime} dz^{\prime} ,$$

where *z* is the direction normal to the interface and *ρ*_c_ is the charge density profile for the *z*-direction. *ψ*(0) on the left-hand side indicates a vacuum for liquid-vapor interfacial systems. Figure 2(f) shows *ψ*(*z*) for water-vapor interfacial systems at *R*_c_ = *L*/2 and Δ = 0.1 nm. The results of the electrostatic potential profile *ψ*(*z*) for water-vapor interfacial systems do not always follow the results of *δ*_*f*_ and *e*_*f*,max_. In comparison with the results of the SPME, the accuracy for estimating *ψ*(*z*) is in the following order: LIPS-SW/FFT (=SPME) > LIPS-5th/FFT = IPSn/DFFT > IPSp/DFFT. However, from Fig. 1(b), the accuracy for estimating *δ*_*f*_ and *e*_*f*,max_ at *R*_c_ = *L*/2 and Δ = 0.1 nm is in the following order: LIPS-5th/FFT > LIPS-SW/FFT > IPSp/DFFT > IPSn/DFFT. There are two possible reasons for this difference. One is the influence of pseudo pair potentials at a relatively short interaction distance. For the pseudo pair potentials, the deviation from the Coulomb potential simply increased with an increase of the interaction distance. In a relatively short interaction distance (*r* < *R*_c_/2), the deviation was in the following order: LIPS-SW < LIPS-5th = IPSn < IPSp. This fact corresponds to the results of *ψ*(*z*), but not to that of *δ*_*f*_ and *e*_*f*,max_ because the deviation at a short interaction distance was concealed in *δ*_*f*_ and *e*_*f*,max_, which reflected the total deviation. The other reason is the influence of the difference between the instantaneous and equilibrated values. *δ*_*f*_ and *e*_*f*,max_ are the instantaneous values calculated using coordinates equilibrated by the SPME, whereas *ψ*(*z*) is the physical property calculated from the molecular structure equilibrated by each method. This difference may affect the evaluation of accuracy. Note that the results of *ψ*(*z*) at *R*_c_ = *L*/2 and Δ = 0.05 nm are almost the same as those plotted in Fig. 2(f), and consistent with the accuracy of *δ*_*f*_ and *e*_*f*,max_ at the same *R*_c_ and Δ conditions (data not shown).

**Figure 3:**
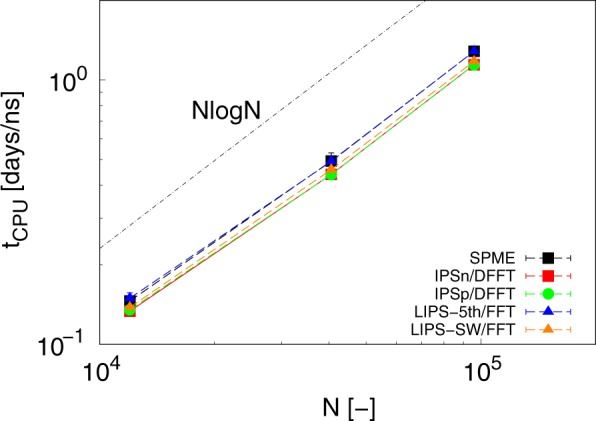
Number of charges *N* dependences on CPU time *t*_CPU_. *O*(*N* log*N*) scaling is also plotted.

To evaluate the computational efficiency of LIPS/FFT, CPU time *t*_CPU_ was measured for water-vapor interfacial systems with different *N*. An Intel(R) Xeon(R) CPU E5-2690 v2 (10 cores/20 threads, 3.00 GHz) was used for the measurement. *L* when *N* = 12000, 40500, and 96000 was 7.2 nm, 10.8 nm, and 14.4 nm, respectively. *R*_c_ and Δ were set to *L*/2 and 0.1 nm, respectively. Figure 3 shows the *N* dependence of *t*_CPU_. The results clearly demonstrate that LIPS/FFT attained almost the same computational efficiency as the SPME. *t*_CPU_ for every method satisfied *O*(*N* log*N*) scaling, which means ideal computational efficiency using the FFT algorithm.

## Conclusion

We developed a combination of the LIPS and FFT, LIPS/FFT, for a substantial advance in the computational efficiency of the LIPS for intermediate-sized systems $(N\sim {10}^{3}\,\mbox{--}\,{10}^{5})$. The performance of LIPS/FFT was evaluated on homogeneous/heterogeneous polar molecular systems. LIPS/FFT attained computational efficiency almost the same as that of the SPME while maintaining the advanced accuracy of the original LIPS. Furthermore, static and dynamic properties calculated by LIPS-SW/FFT was almost the same as that by the SPME. This indicates that LIPS-SW/FFT accurately estimated not only instantaneous values but also equilibrated values. This high accuracy was observed within *R*_c_ ≤ *L*/2. It clearly demonstrated that LIPS/FFT could overcome the concern that the combination of IPS techniques and FFT could not avoid the symmetry effect when requiring large cutoff radii *R*_c_ > *L*/2 for accuracy. One of the potential advantages of IPS techniques is the capability to simulate complex ionic systems that are difficult to accurately simulate using conventional lattice sum methods^79,80,115^. LIPS/FFT can enhance this advantage because it has both high accuracy and computational efficiency, while avoiding the symmetry effect. We conclude that the developed LIPS/FFT has great potential to extend the capability of IPS techniques for the fast and accurate computation of many types of molecular systems that involve highly complex ionic structure and dynamics, such as counterion condensation, ion conduction, and electrochemical migration. In further studies, the capability of LIPS/FFT for complex ionic systems will be intensively evaluated.
